# Acute stress increases ad-libitum alcohol consumption in heavy drinkers, but not through impaired inhibitory control

**DOI:** 10.1007/s00213-016-4205-1

**Published:** 2016-01-27

**Authors:** Elly McGrath, Andrew Jones, Matt Field

**Affiliations:** Department of Psychological Sciences, University of Liverpool, Liverpool, L69 7ZA UK; UK Centre for Tobacco and Alcohol Studies, Liverpool, UK; School of Psychological Sciences, University of Manchester, Manchester, UK

**Keywords:** Alcohol, Disinhibition, Inhibitory control, Stop signal, Stress

## Abstract

**Rationale:**

Stress increases alcohol consumption and the risk of relapse, but little is known about the psychological mechanisms that underlie these effects. One candidate mechanism is inhibitory control, which may be impaired by acute stress and is believed to exert a causal influence on alcohol consumption.

**Objectives:**

We investigated if acute stress would impair inhibitory control and if impaired inhibitory control would be associated with subsequent ad-libitum alcohol consumption in a naturalistic laboratory setting.

**Materials and methods:**

One hundred heavy drinkers took part in an experimental study in a naturalistic ‘bar laboratory’. Participants were randomly assigned to an acute stress (*n* = 50) or control (*n* = 50) group. In the stress group, participants were exposed to the social evaluative threat of giving a self-critical presentation, whereas the control group completed simple anagrams. Prior to and following the manipulation, participants completed the stop signal task as a measure of inhibitory control. Finally, participants completed a bogus taste test, as a measure of ad-libitum alcohol consumption.

**Results:**

The stress manipulation had no effect on performance on the stop signal task. However, there was a small but significant increase in ad-libitum alcohol consumption in the acute stress group compared to that in the control group.

**Conclusions:**

Acute stress increased alcohol consumption in heavy drinkers, in a semi-naturalistic setting. However, this was not through the hypothesised mechanism of a transient impairment in inhibitory control.

**Electronic supplementary material:**

The online version of this article (doi:10.1007/s00213-016-4205-1) contains supplementary material, which is available to authorized users.

## Introduction

The relationships between stress, alcohol consumption, and alcohol use disorders are well documented. The consumption of alcohol is a habitual response to stressful situations in people with alcohol dependence (Marlatt 1996), longitudinal studies suggest a causal relationship between stressful life events and alcohol consumption (Russell et al. 1999), and stress is a strong predictor of problematic drinking and (re)lapse to drinking after a period of abstinence (Noone et al. 1999). Furthermore, both dependent and non-dependent drinkers report that they drink alcohol in order to cope with chronic stress (e.g., financial difficulties) and specific stressful events or challenges (San José et al. 2000; Sinha 2007). These observations are supported by experimental research. Thomas et al. (2011) demonstrated increased ad-libitum alcohol consumption following acute stress in people with alcohol dependence who were not seeking treatment. Similarly, social drinkers voluntarily consume more alcohol immediately after exposure to a psychosocial stressor (de Wit et al. 2003; Magrys and Olmstead 2015), and subjective craving and the subjective value of alcohol increase after a stress challenge in both alcoholics and non-dependent drinkers (Amlung and Mackillop 2014; Field and Powell 2007; Owens et al. 2015).

Very little is known about the psychological mechanism(s) through which stress increases alcohol consumption (Magrys and Olmstead 2015). In the present study, we investigated the possibility that stress may cause transient impairments in inhibitory control, which may in turn influence alcohol consumption (Jones et al. 2013a). Inhibitory control—the ability to stop, change or delay an inappropriate response—is a key component of both impulsivity and executive functioning (Bickel et al. 2012). Impaired impulse control is recognised as a key feature of substance use disorders, including in diagnostic criteria which include the failure to control substance use despite intentions to do so (DSM 5, American Psychiatric Association 2013). Indeed, a recent meta-analysis confirmed a small but robust association between substance abuse (including alcohol use disorders) and impaired inhibitory control when the latter was measured with computerised tasks, such as the stop signal and go/no-go tasks (Smith et al. 2014).

Longitudinal studies suggest that poor inhibitory control plays a causal role in the development and maintenance of alcohol use disorders (Fernie et al. 2013; Nigg et al. 2006; Rubio et al. 2008). Emerging evidence suggests that inhibitory control may function as a ‘state’ that fluctuates in response to internal and environmental cues, and these fluctuations may increase the risk of substance use or (re)lapse (De Wit 2009; Jones et al. 2011b, 2013a). In support of this claim, Weafer and Fillmore (2008) demonstrated that individual differences in inhibitory control when intoxicated predicted voluntary alcohol consumption at a later date. Other studies demonstrated that ‘priming’ of disinhibited or restrained mindsets in sober participants led to short-term increases or decreases in their voluntary alcohol consumption, respectively (Jones et al. 2011a, b). In both of the latter studies, individual differences in inhibitory control were predictive of individual differences in voluntary alcohol consumption.

Momentary stress may be one factor that prompts fluctuations in inhibitory control (Jones et al. 2013a). An earlier model suggested that inhibitory control and emotional regulation of stress responses share common neural substrates (Li and Sinha 2008), such that inhibitory processes are disrupted during and after exposure to acute and chronic stress (Sinha 2001). These models make the shared prediction that acute stress will momentarily impair the ability to control behaviour (Jones et al. 2013a; Li and Sinha 2008). Previous studies that investigated the effects of acute stress on disinhibited behaviour yielded inconclusive findings. Scholz et al. (2009) demonstrated that social evaluative stress impaired performance on the go/no-go task in healthy individuals. In male problem drinkers, acute stress (uncontrollable noise) enhanced the disinhibiting effect of alcohol-related cues on the stop signal task (Zack et al. 2011). However, one study with current and former opiate users demonstrated that acute stress actually improved performance on the go/no-go task (Constantinou et al. 2010).

Our primary objective in the present study was to investigate if acute stress (anticipation of social evaluative threat) would produce a transient impairment in inhibitory control that would be associated with subsequent alcohol consumption. We selected this method of stress induction on the basis of meta-analytic findings that social evaluative threat leads to robust increases in physiological stress response in the laboratory (Dickerson and Kemeny 2004). We made three specific hypotheses: (i) Inhibitory control would be impaired in the stress condition compared to the control condition, (ii) Alcohol consumption would be higher in the stress condition compared to the control condition, and (iii) Impaired inhibitory control would be associated with increased alcohol consumption following the stress manipulation.

## Method

### Participants

One hundred participants (52 female, mean age 20.86 ± 3.93) were recruited from students and staff at the University of Liverpool, using electronic announcements and advertisements placed around campus. Inclusion criteria required participants to be aged over 18 years old and drink in excess of UK government guidelines for safe drinking (14 units per week for women, 21 units for men (Edwards 1996); 1 UK unit = 8 g of pure alcohol). Exclusion criteria included history of alcohol problems, attention deficit hyperactivity disorder, depression- or anxiety-related disorders, all of which were established by self-report when participants were initially screened for participation. The study procedure was approved by the University of Liverpool Research Ethics Committee.

### Materials

#### Stop signal task

Each trial began with a white fixation cross presented in the centre of the screen for 500 ms, immediately followed by presentation of a go stimulus (the letter ‘X’ or the letter ‘O’) for 1000 ms. Participants were instructed to rapidly categorise the go stimulus by pressing one of two keys on the computer keyboard. Go stimuli were uninterrupted on 75 % of trials. The remaining 25 % of trials were stop trials; an auditory tone (the stop signal) was presented shortly after the visual go stimulus. Participants were instructed to inhibit responses to the go stimulus whenever they heard the stop signal. We used a dynamic tracking version of the task (Logan and Cowan 1984); on the very first stop trial, the stop signal occurred 250 ms after presentation of the go stimulus. If participants successfully inhibited their response, this stop signal delay (SSD) increased by 50 ms on the next stop trial, thereby making inhibition more difficult. Whereas if participants failed to inhibit their response, the SSD decreased by 50 ms, which made inhibition easier.

Participants completed a practice block consisting of 12 go trials and four stop trials. Following this, they completed three blocks of 64 trials, each containing 48 go trials and 16 stop trials. The task was programmed using Visual Basic for Windows and was presented on a standard laptop with a 15-in. monitor. The primary dependent measure from the task was the stop signal reaction-time (SSRT), which is an indirect measure of the speed of the inhibitory process (Verbruggen and Logan 2009).

#### Experimental manipulation

Our stress induction manipulation was adapted from one described elsewhere (Gullo and Stieger (2011)). Participants in the stress induction group were instructed to prepare a 5-min presentation on the topic *‘what I dislike about my body and physical appearance’*. Participants were informed that their presentation would be recorded on a video camera and subsequently assessed by a trainee clinical psychologist on the basis of organisation, articulation, openness and defensiveness, in order to gauge their personality. They were given a pen and some paper and told that they had 5 min to plan their presentation, which they would deliver at the end of the experiment. During this time, the experimenter set up a video camera (which was originally hidden from view) before recording the participant saying ‘hello’ and replaying this recording to the participant, in order to strengthen the deception. Participants were never required to perform the presentation. Participants in the control group were given a list of 118 easily solvable anagrams between four and eight letters, and were given 5 min to solve as many as they could (see Field and Powell 2007).

#### Procedure

Participants were informed that the study was an investigation of the relationship between cognitive processes, personality differences and taste perception of alcohol. Participants were randomly allocated to the stress induction or control group, with group allocation stratified by gender. All testing took place within the University of Liverpool ‘bar laboratory’ between 12 and 7 pm. The bar laboratory is modelled on a typical UK bar environment, including beer pumps, posters advertising alcohol and a variety of typical alcoholic beverages on show. After providing informed consent, participants supplied a breath alcohol sample; one male participant provided a positive reading so another session was rescheduled for a later date. All other participants had a breath alcohol level of zero.

Participants completed a questionnaire battery consisting of a retrospective 2-week timeline follow-back diary (Sobell and Sobell 1992) to obtain an estimate of alcohol consumption, the Alcohol Use Disorders Identification Test (AUDIT: Saunders et al. 1993) to examine hazardous drinking, the Temptation and Restraint Inventory (TRI, Collins and Lapp 1992) to examine motivation to limit drinking, and the Barratt impulsivity scale version 11 (BIS, Patton and Stanford 1995) to examine self-reported impulsivity. Self-reported alcohol craving and subjective mood were assessed with the ‘right now’ version of the Approach and Avoidance of Alcohol Questionnaire (AAAQ, McEvoy et al. 2004), and the profile of mood states (POMS, McNair et al. 1992), respectively.

Participants then completed the stop signal task before completing the experimental manipulation (preparing a presentation or solving anagrams, as described above). Following this, participants completed the AAAQ, POMS, a single item VAS scale to measure current thirst, and the stop signal task for a second time (the initial stop signal delay was reset to 250 ms). After completing this, participants in the stress group were told that they would not have to give the presentation (we did this because we were concerned that participants would suppress their alcohol intake if they thought they would have to give a speech in front of camera immediately after the taste test). All participants were then presented with 300 ml of the following beers in unmarked glasses: Becks Vier (4 % ABV), Hoegaarden Wheat Beer (5 % ABV) and Morland Old Golden Hen (4.1 % ABV). They were asked to rate each drink on ten different dimensions (e.g., gassy, pleasant, light) using 10-point Likert scales (see Jones et al. 2011a). All drinks were presented simultaneously, and participants were informed that they could drink as much or as little of each drink as they wished in order to complete the rating scales. They were then left alone for 30 min to taste the beers and complete the rating scales before the glasses and remaining beer were removed, and the total volume of each drink consumed was measured (see Jones et al. 2016).

Finally, participants completed a funnelled debriefing questionnaire that assessed their awareness of the aims and hypotheses of the study. They were first asked an open-ended question that required them to state what they thought the experiment was about. They then completed two multiple-choice questions that captured their awareness of the purpose of the computer task (‘The computer task was designed to…’) and the taste test (‘The purpose of the taste test was to…’). Participants in the stress induction group were also given a multiple-choice question gauging their awareness of the manipulation (‘The purpose of the 5 min presentation was to…’). Variations of these questionnaires have been used in similar studies in our laboratory (Jones et al. 2012, 2013b) to probe awareness of experimental methods in order to control for demand characteristics. Participants were then debriefed before receiving either course credit or £10 in shopping vouchers. Participants were required to remain in the laboratory until their BAL had declined to 0.17 mg/l or below, or they could sign a waiver if they preferred to discharge themselves earlier. The entire experimental session took around 75 min to complete.

#### Data reduction and analysis

Reaction time data was subject to a trimming procedure, similar to that applied in previous studies that used the stop signal task (e.g., Verbruggen and De Houwer 2007). Trials with reaction times faster than 100 ms, slower than 2000 ms and then if more than three standard deviations above the mean were removed prior to analysis. SSRT was analysed using the mean method (Verbruggen and Logan 2009), which involves subtracting mean stop signal delay from mean go reaction time across the three blocks. All stop signal task data from two participants in the control group were lost due to technical problems. Most variables were normally distributed with the exception of two variables from the AAAQ; these were log transformed before analysis to improve their distributions. For the primary dependent variables, we initially included gender as a between-subject factor in all analyses. With the exception of ad-libitum alcohol consumption, there were no significant main effects or interactions involving gender, so ANOVA results are reported collapsed across gender for the remaining variables (mood, craving, and inhibitory control).

## Results

### Group characteristics (see Table 1)

**Table 1:** Group characteristics

	Stress	Control
Age	20.64 ± 4.32	21.08 ± 3.53
AUDIT	14.54 ± 5.35	14.03 ± 4.69
Units cons.	55.76 ± 31.92	62.00 ± 33.28
TRI CEP	21.90 ± 10.08	23.36 ± 10.05
TRI CBC	14.98 ± 8.33	16.82 ± 8.32
BIS attention	18.97 ± 3.02	19.27 ± 2.97
BIS motor	24.50 ± 4.57	24.34 ± 3.90
BIS non-planning	26.78 ± 5.41	27.74 ± 4.98
BIS total	70.25 ± 11.01	71.35 ± 9.14

Values are mean ± SD

*Units cons* number of UK units of alcohol consumed over the previous 2 weeks, *TRI CEP* Temptation and Restraint Inventory cognitive emotional preoccupation subscale, *TRI CBC* Temptation and Restraint Inventory Cognitive Behavioural Concern subscale, *BIS* Barratt impulsivity scale

We examined group differences in age, weekly alcohol consumption, scores on the AUDIT, TRI subscales, and BIS subscales with a multivariate analysis of variance (MANOVA). The overall main effect of the group was not statistically significant (*F*(8, 91) = 0.06, *p* = 0.69), suggesting that the groups were well matched on these variables.

### The effects of stress induction on mood and craving (see Table 2)

**Table 2:** The effects of stress induction on mood and alcohol craving

	Stress		Control	
	Time 1	Time 2	Time 1	Time 2
Tension	5.20 ± 3.00	6.88 ± 4.42	6.46 ± 4.44	5.48 ± 3.01
Anger	1.22 ± 1.63	1.36 ± 3.45	2.60 ± 3.54	2.58 ± 3.70
Vigour	12.94 ± 5.25	10.68 ± 5.93	12.54 ± 5.81	9.86 ± 5.66
AAAQ I-I	4.75 ± 1.71	4.57 ± 1.80	4.66 ± 1.71	4.46 ± 1.96
AAAQ O-C	0.85 ± 1.23	0.87 ± 1.16	0.95 ± 1.17	0.98 ± 1.26
AAAQ R-R	1.18 ± 1.28	0.91 ± 1.12	1.28 ± 1.29	0.97 ± 1.16

Values are mean ± SD

Tension, anger and vigour are subscales from the profile of mood states (POMS)

*AAAQ* approach and avoidance of alcohol questionnaire, *I-I* inclined-indulgent subscale, *O-C* obsessed-compelled subscale, *R-R* resolved-regulated subscale

Subscales from the POMS and AAAQ were analysed using mixed design analysis of variance (ANOVA). A 2 × 2 × 3 ANOVA on POMS subscales with within-subject factors of subscale (3: tension, anxiety, vigour) and time (2: time 1, time 2), and a between-subject factor of group (stress, control) revealed a number of main effects and interactions, all of which were subsumed under a significant three-way interaction subscale *×* time *×* group (*F*(2, 196) = 3.92, *p* = 0.02, *n*_*p*_^2^ = 0.07). To examine the interaction, we ran follow-up 2 × 2 ANOVAs on each subscale separately. For tension, the time *×* group interaction was statistically significant (*F*(1, 98) = 12.88, *p* < 0.01, *n*_*p*_^2^ = 0.12). There were no between-group differences in tension at time 1 (*t*(98) = 1.66, *p* = 0.10), but at time 2, tension ratings were higher in the stress group compared to those in the control group (*t*(98) = 1.85, *p* = 0.03, *d* = 0.37). Within-subject *t* tests confirmed that tension ratings increased from time 1 to time 2 in the stress group (*t*(49) = 2.84, *p* < 0.01, *d* = 0.43), but they decreased over time in the control group (*t*(49) = 2.19, *p* = 0.03, *d* = 0.24). For anger, there was no main effect of time (*F*(1,98) = 0.03, *p* = 0.86) or significant time *×* group interaction (*F*(1,98) = 0.06, *p* = 0.81). For vigour, there was a main effect of time (*F*(1, 98) = 59.05, *p* < 0.01, *n*_*p*_^2^ = 0.38), but no significant time *×* group interaction (*F*(1, 98) = 0.43, *p* = 0.52); all participants reported a reduction in vigour over time, but this was not affected by the experimental manipulation.

A 2 × 2 × 3 ANOVA on AAAQ subscales with within-subject factors of subscale (3: inclined-indulgent, obsessed-compelled, resolved-regulated) and time (2: time 1, time 2), and a between-subject factor of group (stress, control) revealed significant main effects of scale and time, which were subsumed under an interaction (*F*(2, 97) = 4.85, *p* < 0.01, *n*_*p*_^2^ = 0.05). However, there were no main effects or interactions involving experimental group (Fs < 0.01, ps > 0.93). Scores on the inclined-obsessed (*t*(99) = 2.25, *p* = 0.027, *d* = 0.11) and resolved-regulated (*t*(99) = 3.82, *p* < 0.01, *d* = 0.37) subscales tended to decline over time, whereas scores on the obsessed-compelled subscale did not change (*t*(99) = 0.42, *p* = 0.67).

In summary, the stress induction procedure led to a significant increase in self-reported tension ratings, which suggests that the manipulation was successful. Subjective anger, vigour, and alcohol craving (all three subscales of the AAAQ) were unaffected.

### The effects of stress on inhibitory control

SSRT was analysed using a 2 × 2-mixed design ANOVA with a within-subject factor of time (2: time 1, time 2) and a between-subject factor of group (stress, control). There were no significant main effects or interactions: main effect of time (*F*(1, 96) = 1.05, *p* = 0.31), main effect of group (*F*(1, 96) = 0.21, *p* = 0.65), group *×* time interaction (*F*(1, 96) = 0.02, *p* = 0.89). Therefore, acute stress had no effect on inhibitory control.

### The effects of stress on ad-libitum alcohol consumption

**Fig. 1:**
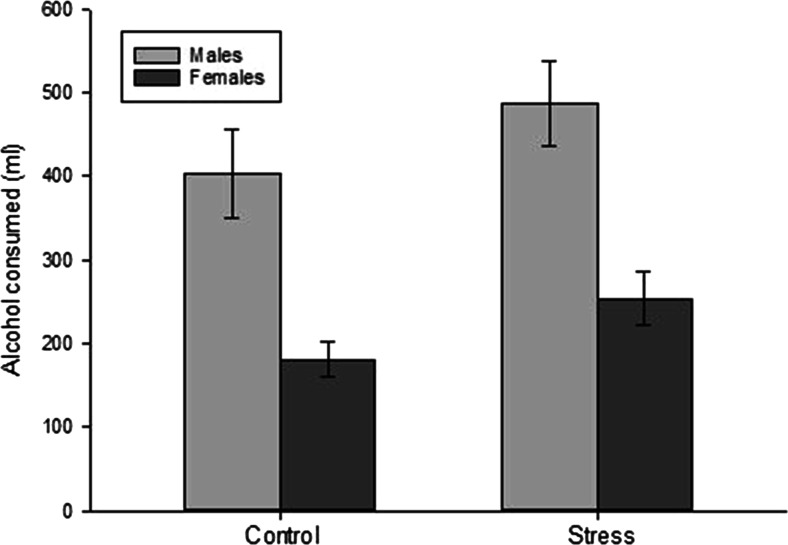
Ad-libitum alcohol consumption in the stress and control groups, split by gender. Values are mean ± SE

Pleasantness ratings for the three drinks did not differ by the group (ts < 0.55, ps > 0.58). Ad-libitum alcohol consumption (the total volume of alcohol consumed, in millilitres; mean = 327 ml; SD = 234; minimum = 15 ml, maximum = 900 ml) was analysed with a univariate ANOVA with factors of the group (stress, control), gender (male, female), and thirst as a covariate, in accordance with previous research using similar forms of the taste test (Houben et al. 2011). There was a significant effect of thirst (*F*(1,95) = 4.20, *p* = 0.04, *n*_*p*_^2^ = 0.04), a main effect of gender (*F*(1,95) = 26.09, *p* < 0.01, *n*_*p*_^2^ = 0.22) with males drinking significantly more than females, and also a main effect of group (*F*(1, 95) = 4.65, *p* = 0.03, *n*_*p*_^2^ = 0.05) with the stress group drinking more than the control group (Fig. 1). The interaction between group *×* gender was not significant (*F*(1, 95) < 0.01, *p* = 0.93).

### Supplementary analyses: awareness

#### Overall awareness

Eleven participants (eight from the stress group, three from the control group) reported awareness of the overall aims of the experiment. Removal of these participants had no effect on SSRT: the main effect of time (*F*(1,85) = 0.83, *p* = 0.37) and the time *×* group interaction (*F*(1,85) = 0.16, *p* = 0.69) remained non-significant. Their removal also had no effect on alcohol consumption, as the main effect of the group remained significant (*F*(1, 84) = 4.24, *p* = 0.04, *n*_*p*_^2^ = 0.05).

#### Awareness of the taste test

Fifty-two participants (52 % of sample, identical proportions in the stress and control groups) demonstrated awareness of the true purpose of the taste test. To examine whether awareness of the taste test moderated alcohol consumption, we performed a univariate ANOVA with between-subject factors of group (stress induction, control), gender (male, female) and awareness (aware, unaware), with thirst as a covariate. The main effect of the group was still significant (*F*(1,91) = 4.80, *p* = 0.03, *n*_*p*_^2^ = 0.05). However, there was no main effect of awareness (*F*(1,91) = 0.99, *p* = 0.32) or any interactions involving awareness, gender or group (Fs < 1.02, ps > 0.32).

#### Awareness of the stress manipulation (see also Supplemental online materials)

In the stress group, 32 participants (64 %) reported awareness of the purpose of the experimental manipulation. Therefore, we re-ran the main analyses using the three groups (stress aware, stress unaware and control). There were no effects of stress awareness on AAAQ scores or SSRT (see Supplemental online materials for details). A 2 × 3 × 3 ANOVA on POMS subscales with within-subject factors of time (2: time 1, time 2), subscale (3: tension, anxiety, vigour), and a between-subject factor of group (3: stress aware, stress unaware, control) indicated a significant scale *×* time *×* group interaction (*F*(4, 194) = 4.01, *p* < 0.01, *n*_*p*_^2^ = 0.08). Within-subject *t* tests confirmed that tension increased from time 1 to time 2 only in the stress unaware group (*t*(17) = 2.79, *p* = 0.01, *d* = 0.75), but not in the stress aware or control groups (ps > 0.1).

Ad-libitum alcohol consumption was analysed using a univariate ANOVA with between-subject factors of group (stress aware, stress unaware, control) and gender (male, female) and thirst as a covariate. There was a significant effect of thirst (*F*(1,93) = 4.73, *p* = 0.03, *n*_*p*_^2^ = 0.05), a main effect of gender (*F*(1, 93) = 18.43, *p* < 0.01, *n*_*p*_^2^ = 0.17) and a main effect of group (*F*(2,93) = 3.42, *p* = 0.04, *n*_*p*_^2^ = 0.07). The stress unaware group (469.67 ml ± 277.99) consumed significantly more alcohol than both stress aware (306.91 ml ± 194.39; (*t*(48) = 2.43, *p* = 0.02, *d* = 0.70) and control groups (288.14 ml ± 224.15; *t*(66) = 2.76, *p* < 0.01, *d* = 0.70), but the stress aware and control groups did not differ (*t*(80) = 0.39, *p* = 0.70).

To summarise, the stress manipulation increased tension and ad-libitum alcohol consumption only in the subgroup of participants who reported no awareness of the purpose of the experimental manipulation.

### Correlations between SSRT, craving, mood and ad-libitum consumption

We investigated correlations (Pearsons, two-tailed) between SSRT, AAAQ and POMS subscales at time 2 and ad-libitum consumption during the taste test in each group using a conservative *p* value (*p* < 0.01) to correct for multiple correlations. In the stress group, there were no significant correlations between POMS subscales and ad-libitum consumption (rs < 0.16, ps > 0.28). Scores on both the AAAQ inclined (*r* = 0.54, *p* < 0.01) and obsessed (*r* = 0.37, *p* < 0.01) subscales were significantly associated with ad-libitum consumption. In the control group, there was a significant correlation between POMS vigour (*r* = 0.48, *p* < 0.01) and ad-libitum consumption. Similarly, there was a significant correlation between AAAQ inclined and ad-libitum consumption (*r* = 0.46, *p* < 0.01). All other correlations involving POMS and AAAQ were non-significant. Neither group demonstrated a significant association between SSRT and ad-libitum consumption (rs < 0.17, ps > 0.29). Examining associations in the stress aware and unaware groups separately did not significantly influence these results (see Supplemental materials).

## Discussion

Results from this study demonstrated that acute stress did not lead to impairments in inhibitory control in heavy drinkers who were tested in a semi-naturalistic ‘bar lab’. However, we demonstrated that stress increased ad-libitum alcohol consumption, relative to a control manipulation.

We found no support for our primary hypothesis, that acute stress would impair inhibitory control. The absence of any effect of stress on inhibitory control is surprising given theoretical predictions that emotional regulation of stress responses and inhibitory control compete for resources (Li and Sinha 2008), and previous findings that acute stress and alcohol-related cues reliably impaired inhibitory control in male problem drinkers (Zack et al. 2011). However, closer examination of the existing literature suggests that these inconsistencies could be attributable to heterogeneity of the stress response: the effect of stress on executive functioning may be ‘u-shaped’, with high and low stress impairing executive function, but moderate stress improving it (Henderson et al. 2012). Furthermore, many factors may moderate the response to stress (Biondi and Picardi 1999). For example, Scholz et al. (2009) demonstrated that the effects of stress on inhibitory control could be mitigated if participants adopted deliberate strategies. We cannot rule out that the possibility that participants in the present study may have adopted strategies to preserve their performance on the stop signal task, and this is an issue that warrants further investigation.

The observed increase in ad-libitum alcohol consumption in the stress group relative to the control group is consistent with observations that stress contributes to heavy drinking and relapse after abstinence (San José et al. 2000; Sinha 2007), and with findings from earlier laboratory studies (De Wit et al. 2003). However, we found no support for our hypothesis that individual differences in inhibitory control would be associated with ad-libitum alcohol consumption. This is inconsistent with predictions made by recent theoretical models (De Wit 2009; Jones et al. 2013a) and represents a failure to replicate previous findings (Jones et al. 2011a, b; Petit et al. 2012; Weafer and Fillmore 2008).

Our study has limitations. First, in contrast to earlier findings (e.g., Amlung and MacKillop 2014), our stress manipulation had no effect on subjective craving. This could be because testing took place in a ‘bar lab’, which may have masked the effects of stress (see Field et al. 2008). Alternatively, participants completed the stop signal task twice during the study, and the high working memory load imposed by this task could have suppressed subjective craving (see Van Dillen et al. 2013). Second, the brief interval between assessments of subjective mood (either side of the stress induction/control manipulations) could have contributed to the high level of participant awareness of the purpose of the stress induction manipulation, and their awareness clearly influenced their subjective mood and alcohol consumption. Future studies could use more objective measures of the stress response such as cortisol (Hellhammer et al. 2009) in order to reduce participant awareness and increase the effectiveness of the stress manipulation. However, we note that our multiple-choice measure of participant awareness for the purpose of the stress induction may have yielded an inflated estimate of the number of participants who were aware, because when participants were asked an open-ended question about the purpose of the study, only a minority (eight) demonstrated awareness. Furthermore, participants’ awareness of the purpose of the stress manipulation should not be confused with demand characteristics, because the stress manipulation only affected mood and alcohol consumption in participants who were *not* aware of the purpose of the manipulation. Nevertheless, in order to overcome these limitations, future studies should attempt to disguise the purpose of the stress manipulation and assess awareness of its purpose using more subtle measures.

Our findings suggest interesting avenues for future research. First, we selected our stress manipulation on the basis of a meta-analysis that demonstrated that social evaluative threat leads to robust increases in physiological stress response (Dickerson and Kemeny 2004). However, earlier studies used different types of stress manipulations such as personalised imagery (Sinha 2009) or challenging mental arithmetic tasks combined with social evaluative threat in which participants actually gave a speech that was videotaped (de Wit et al. 2003). Therefore, caution is required when comparing findings across studies. Second, our study was conducted in a ‘bar laboratory’ which mimics the context in which alcohol is normally consumed. Our findings are consistent with those from other studies that were conducted in conventional laboratory settings (e.g., de Wit et al. 2003; Magrys and Olmstead 2015), and further research is required to investigate if contextual cues and acute stressors have independent or additive effects on alcohol consumption. Third, it is important to investigate if effects of acute stress on inhibitory control can be detected by alternative measures, such as the go/no-go task rather than the stop signal task that we used in the present study (see Constantinou et al. 2010). Finally, it is important to investigate alternative mechanisms through which acute stress increases alcohol consumption. For example, given that acute stress increases attentional biases for alcohol cues (e.g., Field and Powell 2007), and that discrete alcohol cues lead to transient impairments in inhibitory control (Jones & Field, 2015), an extension of this study might involve embedding alcohol-related and neutral cues into a stop signal task in order to test the prediction that stress would impair inhibitory control, but only when discrete alcohol-related cues are present (see Zack et al. 2011).

To conclude, we found that acute stress increased alcohol consumption in sober heavy drinkers, but this was not through the hypothesised mechanism of transient changes in inhibitory control. These results are inconsistent with theoretical predictions that stress can cause transient changes in inhibitory control, and that these changes contribute to increased alcohol consumption.

## Electronic supplementary material

ESM 1 (DOCX 18 kb)
