# Supplementary material for: Acute stress increases ad-libitum alcohol consumption in heavy drinkers, but not through impaired inhibitory control
Source: Psychopharmacology (Berl). 2016 Jan 27;233:1227–34. doi: 10.1007/s00213-016-4205-1 (PMC4801987; doi:10.1007/s00213-016-4205-1)
Supplement: Supplementary file 1 — (DOCX 18 kb) [file 213_2016_4205_MOESM1_ESM.docx]

*Supplementary group characteristics*

Group characteristics (stress aware, stress unaware, control) in age, weekly alcohol consumption scores on the AUDIT, TRI subscales, and BIS subscales were investigated with a Multivariate Analysis of Variance (MANOVA). The main effect of group was not significant, suggesting groups were well matched on these variables F(14, 184) = 0.75, p = 0.73).

*Supplemental analysis of stress awareness on inhibitory control*

SSRT was analysed using a 2 x 3 mixed ANOVA with a within-subjects factor of time (2: time 1, time 2) and a between subjects factor of group (3: stress aware, stress unaware, control). There were no significant main effects or interactions: main effect of time (F(1, 95) = 0.35, p = .55) main effect of group (F(2, 96) = 0.32, p = .73), group x time interaction (F(1, 96) = 0.78, p = .46).

*Supplemental analysis of stress awareness on craving*

A 2 x 3 x 3 ANOVA on AAAQ subscales with within-subject factors of subscale (3: inclined-indulgent, obsessed-compelled, resolved-regulated) and time (2: time 1, time 2), and a between-subjects factor of group (stress aware, stress unaware, control) revealed significant main effects of scale and time, which were subsumed under an interaction (F(2, 96) = 4.34, p = .01, *n_p_^2^* = .04). However, there were no main effects or interactions involving experimental group (Fs < .43, ps > .91).

*Correlations between SSRT, craving, mood and ad-libitum consumption*

We investigated Pearson’s correlations between SSRT, AAAQ and POMS subscales at time 2 and *ad-libitum* consumption during the taste test in stress aware and unaware groups separately using a conservative p-value (p < .01) to correct for multiple correlations. The only significant correlation at this level was between AAAQ inclined – obsessed and *ad-libitum* consumption in the stress aware group (r = .54, p < .01). Note that the correlation between AAAQ inclined and ad-libitum consumption in the stress unaware group was also strong (r = .49, p = .02), but may not have reached the p < .01 threshold due to lower statistical power in this group (N=18).

Supplemental table 1: Craving, Mood and SSRT scores split separately by individuals who showed awareness of the stress manipulation. Values are means ± SDs

Stress Aware (N=32) Stress unaware (N=18)

Time 1 Time 2 Time 1 Time 2

Tension 5.72 ± 3.10 6.69 ± 4.08 4.28 ± 2.65 7.22 ± 5.09

Anger 1.25 ± 1.74 1.28 ± 4.07 1.17 ± 1.47 1.50 ± 2.04

Vigour 12.69 ± 5.23 11.16 ± 5.40 13.39 ± 5.41 9.83 ± 6.84

AAAQ I-I 4.47 ± 1.74 4.31 ± 1.73 5.24 ± 1.59 5.02 ± 1.87

AAAQ O-C 0.65 ± 0.74 0.68 ± 0.83 1.21 ± 1.78 1.19 ± 1.57

AAAQ R-R 1.03 ± 1.13 0.87 ± 0.96 1.46 ± 1.50 0.99 ± 1.39

SSRT 208.31 ± 36.25 216.24 ± 31.84 221.26 ± 39.22 216.08 ± 39.22

Tension, Anger and Vigour: subscales from the Profile of Mood States (POMS)

AAAQ: Approach and Avoidance of Alcohol Questionnaire; I-I = Inclined-Indulgent subscale; O-C = Obsessed-Compelled subscale; R-R = Resolved-Regulated subscale

SSRT: Stop signal reaction time.
